# Association between falls and cognitive performance among community-dwelling older people: a cross-sectional study

**DOI:** 10.1590/1516-3180.2021.0180.R1.15092021

**Published:** 2022-03-18

**Authors:** Isabella Vittoria Fallaci, Daiene de Morais Fabrício, Tiago da Silva Alexandre, Marcos Hortes Nisihara Chagas

**Affiliations:** I BSc. Gerontologist, Department of Gerontology, Research Group on Mental Health, Cognition and Aging, Universidade Federal de São Carlos (UFSCar), São Carlos (SP), Brazil.; II MSc. Gerontologist and Doctoral Student, Department of Gerontology, Research Group on Mental Health, Cognition and Aging, Universidade Federal de São Carlos (UFSCar), São Carlos (SP), Brazil.; III PhD. Physiotherapist and Associate Professor, Department of Gerontology, Universidade Federal de São Carlos (UFSCar), São Carlos (SP), Brazil.; IV MD, PhD. Psychiatrist and Associate Professor, Department of Gerontology, Research Group on Mental Health, Cognition and Aging, Universidade Federal de São Carlos (UFSCar), São Carlos (SP), Brazil; Associate Professor, Department of Gerontology, Universidade Federal de São Carlos (UFSCar), São Carlos (SP), Brazil; and Technical Director, Medical Residency Program on Psychiatry, Instituto Bairral de Psiquiatria, Itapira (SP), Brazil.

**Keywords:** Cognition, Mental health, Accidental falls, Falls, Older people, Mental hygiene

## Abstract

**BACKGROUND:**

Falls among older people have a negative impact on health and therefore constitute a public health problem. Cognitive decline can also accompany the aging process, and both conditions lead to significant increases in morbidity and mortality in this population.

**Objective:**

To analyze the cognitive performance of older people, classified as non-fallers, sporadic fallers and recurrent fallers, and investigate the relationship between falls and cognition.

**DESIGN AND SETTING:**

Cross-sectional study conducted in the interior of the state of São Paulo, Brazil.

**METHODS:**

Evaluations on 230 older people were conducted. They were divided into three groups: non-fallers, sporadic fallers (one fall) and recurrent fallers (two or more falls). The Mini-Mental State Examination, Consortium to Establish a Registry for Alzheimer’s Disease (CERAD), Brief Cognitive Screening Battery (BCSB), Cambridge Examination for Mental Disorders of the Elderly (CAMDEX) similarities subtest and digit span test were applied.

**RESULTS:**

In multinomial logistic regression, being a recurrent faller was significantly associated with lower scores in the CERAD word list (odds ratio, OR = 0.92; 95% confidence interval, CI, 0.86-0.98; P = 0.01), in CERAD constructive praxis (OR = 0.88; 95% CI, 0.79-0.98; P = 0.02), in BCSB figure list memory (OR = 0.94; 95% CI, 0.89-0.99; P = 0.02) and in verbal fluency (OR = 0.89; 95% CI, 0.81-0.97; P = 0.01). Recurrent fallers also had lower scores in these same tests, compared with sporadic fallers.

**CONCLUSION:**

Cognitive impairment, especially in the domains of memory and executive functioning, can influence occurrences of recurrent falls.

## INTRODUCTION

Occurrence of falls among older people constitutes a public health problem due to their negative impact on quality of life in this population. Falls can contribute to loss of independence, social isolation, institutionalization and mortality.^1^ The World Health Organization (WHO)^2^ defines a fall as “inadvertently coming to rest on the ground, floor or other lower level, excluding an intentional change in position to rest on furniture, a wall or other objects”.^2^

The prevalence of falls among older Brazilians ranges from 10 to 35%, depending on the region analyzed.^3^ Falls may occur due to osteoarticular and/or neurological decline related to the aging process or due to an adverse clinical condition that affects mechanisms of balance and stability.^4^ The risk factors for falls may be intrinsic or extrinsic. Intrinsic factors comprise characteristics or clinical factors relating to older people, such as dizziness, weakness and chronic health conditions.^5^ Extrinsic factors include characteristics of the surrounding environment, such as uneven surfaces and inadequate lighting.^5^

Like falls, cognitive impairment can significantly increase morbidity and mortality among older people. Indeed, these two events often coexist in this population and contribute to significantly increased healthcare expenditures and reduction of quality of life. It has also been reported that individuals with cognitive decline exhibit gait deficits.^6,7^ Thus, altered cognitive capacity may have a negative impact on postural stability, thus leading to greater risk of falls.^6^

Despite the evidence accumulated to date, the mechanisms involved in the association between cognitive impairment and the risk of falls have not yet been fully clarified.^6^ These two conditions have always been addressed as distinct geriatric syndromes, which hinders the understanding of cognitive-motor interactions.^8^ Studies have indicated that cognitive decline, specifically with regard to attention and executive functions, can compromise gait and contribute to occurrences of falls in the older population.^9,10^ Moreover, the prevalence of gait disorders is higher among older people with major neurocognitive disorder (MND), and this impairment increases as cognitive performance diminishes.^11^

A prospective population-based study conducted among 9,279 community-dwelling elderly people sought to investigate whether their experience of falls in the past two years was responsible for cognitive function, after adjusting for all possible confounding variables.^12^ The results from this study demonstrated that elderly people with an experience of falling had a cognitive performance estimate that was 0.13% lower (95% confidence interval, CI: 0.023 to 0.002; P-value: 0.017) than the estimate for those without a fall experience.^12^ Hence, evaluating the cognitive performance of elderly fallers becomes essential. Complementarily, it was sought in another study to assess cognitive function and its relationships with balance, history of falls and fear of falling, among 250 elderly people.^13^ Elderly people with cognitive decline were found to have greater fear of falling than elderly people without cognitive decline (P = 0.008).^13^ These results corroborate the importance of evaluating the presence of new falls in this population.

Few studies have specifically investigated the most altered cognitive domains in the presence of falls. A cross-sectional study involving 462 older people investigated the association between cognitive capacity and falls and found that the prevalence of falls among those with cognitive impairment evaluated using the Mini-Mental State Examination was 42%, which confirmed the strong association between these variables.^11^ Thus, a more comprehensive assessment of altered cognitive domains among older people with a history of falls may contribute towards planning interventions aimed at preventing occurrences of both of these negative outcomes in specific domains, to enable maintenance of physical and cognitive health throughout the aging process.

Some studies have addressed the importance of assessing the frequency of falls, considering that the number of falls may predict greater health risks.^14^ A study evaluating 325 community-dwelling older people who suffered at least one fall in the previous year found that greater numbers of falls were associated with higher frequency of risk factors, such as fractures, loss of mobility due to the fear of falling again and hospitalizations. These were considered to be predictors of reduced quality of life of this population.^14^

In this light, assessment of cognitive performance and numbers of falls in the older population can help broaden our understanding of the association between falls and cognition and may assist in planning early interventions.

## OBJECTIVE

Therefore, the aim of the present study was to analyze cognitive performance among older people classified as non-fallers, sporadic fallers and recurrent fallers.

## METHODS

### Setting and participants

This study was conducted in the city of São Carlos, in the state of São Paulo, Brazil. This city has 28,696 residents aged 60 years or older, corresponding to 12.92% of the total population.^15^ The study was conducted in the area of coverage of one family health unit: in this area there were 317 residents aged 60 years and older, according to data from that health unit. There was no potential difference in this area in relation to other regions of the city. The exclusion criteria were situations of diagnoses of MND, severe mental disorders or intellectual disability. In addition, potential subjects were excluded if they had any other serious health problem that made it impossible for them to respond to the tests.

During home visits, 28 individuals were not encountered at their homes or were found to no longer live at the address, five declined to participate in the study, two were bedridden and 23 did not answer the questionnaire addressing falls. Furthermore, 25 elderly people who had been diagnosed with MND, two with schizophrenia and two with intellectual disabilities were excluded. Thus, 230 older people were included in the present study. Data were collected between March 2016 and February 2017.

### Groups

The individuals were allocated to different groups based on the numbers of falls, which were investigated using the following question: “How many falls have you suffered in the last 12 months?” Depending on the answer to this question, the participants were classified as non-fallers (those who had not suffered any falls in the previous year), sporadic fallers (those who had suffered a single fall in the previous year) or recurrent fallers (those who had suffered two or more falls in the previous year). During the interviews, a companion of the elderly subject was always present. For example, this could be someone who lived together with this subject (husband or wife or children).

### Cognitive assessment

The cognitive assessment was performed by means of the following battery of tests:

- Mini-Mental State Examination (MMSE): This is a widely used screening tool for evaluating overall cognition, with scores ranging from 0 to 30 points, which assesses temporal and spatial orientation, memory (registration and recall), language, attention and calculation.^16^
- Consortium to Establish a Registry for Alzheimer’s Disease (CERAD): This battery is composed of the following tests: verbal fluency (animal naming), Boston naming (15 items), recall of a list of words, constructional praxis, recognition list and recall of praxis. In the evaluation of memory, a list of ten words is presented to the participant, who is asked to remember as many words as possible within a maximum recall time of 90 seconds (free recall). The procedure is repeated two more times and the score is obtained as the sum of the words recalled during the three trials. Constructional praxis is evaluated through copying four figures. Delayed recall of the list of words presented previously is then performed for a maximum of 90 seconds. Next, the initial ten words are presented together with ten distractors and the participant is asked to recognize which words were in the original list. Lastly, the participant is asked to reproduce the four drawings that had previously been copied.^17^
- Brief Cognitive Screening Battery (BCSB): This battery involves verbal fluency (animals), a clock drawing test and a figure memory test (incidental, immediate recall, learning, delayed recall and recognition). The BCSB has been shown to have good accuracy for populations with high rates of illiteracy or low levels of schooling. Memory is assessed through presentation of ten figures (frog, spoon, comb, tree, turtle, key, airplane, house, book and bucket), which the participants are asked to name immediately. The figures are then presented two more times, followed by immediate recall. After the verbal fluency test and the clock drawing test (both of these form part of the BCSB), the participant is asked to remember the ten figures (delayed recall). Lastly, there is a recognition test (consisting of the ten target figures plus ten distracting figures).^18^
- Similarities subtest of the Cambridge Mental Disorders of the Elderly Examination (CAMDEX): This test consists of four questions to assess the abstraction capacity of the participant, based on the similarity between two things or objects; for example: What do an apple and banana have in common?^19^
- Digit span test (forward and backward): This test is composed of seven pairs of numerical sequences with different quantities of digits applied in forward and backward order. The sequences have three to nine numbers in the forward order and two to eight numbers in the backward order. The test ends after the participants err in two consecutive sequences.^20^ The maximum quantity of numbers repeated without error is recorded for each version (forward and backward).

### Procedures

This study received approval from the institutional review board of the Federal University of São Carlos (October 29, 2015; certificate number: 48602515.5.0000.5504). All the volunteers who agreed to participate in the study signed a statement of informed consent prior to the interviews.

Five trained gerontologists conducted the interviews in the participants’ homes, during which sociodemographic and clinical data were collected and the cognitive tests were applied. The presence of polypharmacy was also assessed, and was defined as the use of five or more medications.^21^ Information relating to medical conditions (histories of stroke, diabetes mellitus, hypertension, heart disease and dyslipidemia) was obtained through self-reports. The evaluation took 60 to 90 minutes. Furthermore, these participants were evaluated within a maximum of 30 days by three psychiatrists, who performed a diagnostic evaluation based on the criteria of the Diagnostic and Statistical Manual of Mental Disorders, 5^th^ edition (DSM-5).

### Statistical analysis

Descriptive analysis was performed on the variables, considering the overall sample and the three fall classification groups (non-fallers, sporadic fallers and recurrent fallers). The chi-square test, ANOVA or the Kruskal-Wallis test was used to compare differences among the groups, depending on the distribution of the sample and the type of variable. Associations between the groups of fallers and cognitive performance were analyzed using multinomial logistic regression. Variables for which statistically significant differences were found between groups were adjusted in the regression model. The odds ratio (OR) and its respective 95% confidence interval (95% CI) were calculated for each cognitive test. The variance inflation factor (VIF) was used to detect multicollinearity. All VIF values were found to be less than 2, which showed that multicollinearity did not affect the model. All analyses were conducted with the aid of the Statistical Package for the Social Sciences (SPSS), version 21.0 (International Business Machines Corp., Armonk, New York, United States). The significance level was set at 5% (P ≤ 0.05).

## RESULTS

The 230 older people included in this study were classified as non-fallers (n = 159), sporadic fallers (n = 38) or recurrent fallers (n = 33). The sociodemographic and clinical characteristics of the groups are displayed in Table 1. A significant difference among the groups was found with regard to the occurrence of polypharmacy (P = 0.03).

**Table 1.** Sociodemographic and clinical variables of the total sample and of the three fall groups

Variables	Total (n = 230)	Non-fallers (n = 159)	Sporadic fallers (n = 38)	Recurrent fallers (n = 33)	P
	Mean (standard deviation)				
**Age**	69.74 (± 7.30)	69.25 (± 6.97)	70.45 (± 8.46)	71.27 (± 7.38)	0.36
**Years of schooling**	3.39 (± 3.12)	3.68 (± 3.36)	2.87 (± 2.34)	2.64 (± 2.52)	0.29
**Percentage**					
**Illiterate**					
Yes	23.8%	23.6%	23.8%	25%	0.98
No	76.2%	76.4%	76.2%	75%	
**Sex**					
Female	59.1%	57.2%	63.2%	63.6%	0.68
Male	40.9%	42.8%	36.8%	36.4%	
Retired					
Yes	72.2%	74.2%	71.1%	63.6%	0.46
No	27.8%	25.8%	28.9%	36.4%	
**Marital status**					
Married/with partner	60.4%	64.8%	52.6%	48.5%	0.12
**Polypharmacy**					
Yes	29.6%	25.2%	47.4%	30.3%	0.03*
No	70.4%	74.8%	52.6%	69.7%	
**Systemic arterial hypertension**					
Yes	61.3%	58.5%	65.8%	69.7%	0.40
No	38.7%	41.5%	34.2%	30.3%	
**Dyslipidemia**					
Yes	29.1%	28.3%	34.2%	27.3%	0.75
No	70.9%	71.7%	65.8%	72.7%	
**Cardiac disease**					
Yes	18.3%	16.4%	18.4%	27.3%	0.34
No	81.7%	83.6%	81.6%	72.7%	
**Stroke**					
Yes	9.1%	9.4%	10.5%	6.1%	0.79
No	90.9%	90.6%	89.5%	93.9%	
**Arthritis**					
Yes	22.6%	19.5%	26.3%	33.3%	0.19
No	77.4%	80.5%	73.7%	66.7%	
**Current smoker**					
Yes	12.2%	13.8%	5.3%	12.1%	0.35
No	87.8%	86.2%	94.7%	87.9%	

*Significant at P < 0.05.

The multinomial logistic regression results are shown in Table 2. After adjusting for polypharmacy, the regression data indicated that being a recurrent faller was significantly associated with lower scores in the CERAD word list (OR = 0.92; 95% CI 0.86-0.98; P = 0.01), in CERAD constructive praxis (OR = 0.88; 95% CI 0.79-0.98; P = 0.02), in BCSB figure list memory (OR = 0.94; 95% CI 0.89-0.99; P = 0.02) and in verbal fluency (OR = 0.89; 95% CI 0.81-0.97; P = 0.01), compared with non-fallers. In addition, being a recurrent faller was significantly associated with lower scores in the CERAD word list (OR = 0.89; 95% CI 0.82-0.96; P = 0.01), in CERAD constructive praxis (OR = 0.87; 95% CI 0.76-1.00; P = 0.05), in BCSB figure list memory (OR = 0.93; 95% CI 0.87-1.00; P = 0.05) and in verbal fluency (OR = 0.87; 95% CI 0.77-0.98; P = 0.02), compared with sporadic fallers.

**Table 2.** Multinomial regression analysis of cognitive domains in relation to each group of falls

Variables	Groups					
	Sporadic fallers versus non-fallers (reference)		Recurrent fallers versus non-fallers (reference)		Recurrent fallers versus sporadic fallers (reference)	
	OR (95% CI)	P	OR (95% CI)	P	OR (95% CI)	P
MMSE	1.02 (0.95-1.10)	0.61	0.95 (0.88-1.01)	0.11	0.98 (0.91-1.06)	0.10
Word list memory (CERAD)	1.04 (0.98-1.10)	0.25	0.92 (0.86-0.98)	0.01**	0.89 (0.82-0.96)	0.01**
Delayed recall (CERAD)	1.06 (0.93-1.22)	0.38	0.97 (0.85-1.12)	0.70	0.92 (0.77-1.09)	0.32
Recognition (CERAD)	1.07 (0.95-1.21)	0.28	0.98 (0.87-1.09)	0.67	0.91 (0.77-1.06)	0.23
Constructional praxis (CERAD)	1.01 (0.90-1.12)	0.90	0.88 (0.79-0.98)	0.02*	0.87 (0.76-1.00)	0.05*
Figure list memory (BCSB)	1.01 (0.95-1.06)	0.83	0.94 (0.89-0.99)	0.02*	0.93 (0.87-1.00)	0.05*
Delayed recall (BCSB)	1.02 (0.90-1.17)	0.73	0.90 (0.80-1.01)	0.09	0.88 (0.75-1.03)	0.11
Recognition (BCSB)	1.15 (0.97-1.36)	0.11	0.93 (0.83-1.04)	0.20	0.81 (0.67-0.98)	0.38
Abstraction subtest (CAMDEX)	1.04 (0.89-1.20)	0.64	0.96 (0.82-1.12)	0.61	0.93 (0.76-1.13)	0.44
Clock drawing test	1.03 (0.94-1.13)	0.56	0.92 (0.83-1.02)	0.12	0.90 (0.79-1.02)	0.09
Verbal fluency	1.02 (0.93-1.12)	0.65	0.89 (0.81-0.97)	0.01**	0.87 (0.77-0.98)	0.02*
Boston naming test	1.08 (0.95-1.23)	0.22	0.96 (0.85-1.07)	0.45	0.88 (0.76-1.03)	0.12
Digit extension test (forward)	0.94 (0.74-1.20)	0.63	0.85 (0.66-1.08)	0.18	0.90 (0.66-1.23)	0.50
Digit extension test (backward)	1.20 (0.91-1.60)	0.20	0.98 (0.74-1.29)	0.88	0.82 (0.57-1.16)	0.26

OR = odds ratio; CI = confidence interval; MMSE = Mini-Mental State Examination; CERAD = Consortium to Establish a Registry for Alzheimer’s Disease; BCSB = Brief Cognitive Screening Battery; CAMDEX = Cambridge Examination for Mental Disorders of the Elderly; *significant at P < 0.05; **significant at P < 0.01.

## DISCUSSION

In the present study, we evaluated the cognitive performance of older people in relation to their numbers of falls. We found that cognitive impairment could influence the number of falls, considering that statistically significant differences were found between non-fallers/sporadic fallers and recurrent fallers regarding the cognitive domains of immediate recall, praxis (visuospatial skills) and executive functions (verbal fluency). The group of recurrent fallers performed more poorly in these domains. It was also noteworthy that no significant differences were found between the non-fallers and sporadic fallers regarding any of the cognitive tests applied.

Investigation of specific cognitive domains according to the number of falls has been little explored in the recent literature. The current evidence sustains the notion that compromised executive functions may predict occurrences of falls.^22^ In the present study, recurrent fallers performed worse in the tests that evaluated this domain, even after adjusting for polypharmacy. Hsu et al.^22^ conducted a review of the literature to identify cognitive domains associated with the risk of falls among older people and found that 12 studies reported an association between executive functions and the risk of falls, whereas only three studies did not find such an association. These authors suggested that changes to gait might be usable as markers of cognitive decline.

Using methods similar to those in the present study, Holtzer et al.^23^ analyzed the association between cognition and falls among older people, who were classified as single (sporadic) fallers or recurrent fallers, based on self-reported falls. A neuropsychological battery was used to assess executive functions, processing speed, attention and memory. The regression analyses revealed that an increase in the standard deviation of the attention and processing speed tests was associated with an approximately 50% reduction in the risk of falls. Lower executive functioning scores were associated with an increase in falls only in the group of recurrent fallers. These findings are concordant with the results from the present study, which revealed differences between sporadic fallers and recurrent fallers. In contrast, Holtzer et al.^23^ found that there was no association between the memory domain and an increased risk of falls, either among single fallers or among recurrent fallers.

Studies investigating the association between cognition and falls among older people have generally not found any increased risk of falls in relation to declining memory.^23–26^ Anstey et al.^24^ and Herman et al.^25^ reported that executive functions, processing speed (executive functioning) and visuospatial skills (constructional praxis) were the main predictors of falls, which is in agreement with the present findings. However, memory decline was also found in the group of recurrent fallers in the present study, but not in the other two groups. Likewise, Al-Sari et al.^27^ found that subjective memory complaints might predict fall events among older women. In that study, self-reported complaints of forgetfulness were seen to be associated with increased risk of more restricted mobility, as well as a greater risk of falls and fractures.^27^ In another study, it was found that participants with episodic memory impairment were at 24 to 29% greater risk of falls than those without impairment, over an eight-year follow-up period.^28^ In a prospective study, it was also found that deficits in immediate recall and diminished verbal capacity and processing speed were associated with increased risk of falls.^24^

Concerning the divergent results relating to memory, there is the possibility that non-amnestic cognitive impairment can co-occur with amnestic deficits.^29^ Moreover, memory decline directly affects one’s correct recall regarding the number of falls in the previous year.^30^ Thus, reporting bias may occur when investigating the association between falls and memory among older people, considering that falls are self-reported in the majority of studies.

Recurrent falls present as a chronic disorder. Risk factors that are not previously resolved and identified can result in recurrent falls and negatively affect the quality of life of elderly people.^31^ Therefore, it is important to consider the usefulness of investigating some risk factors, such as histories of falls, injuries and comorbidities among elderly people who have already fallen at some point. Within primary care, screening for visual, balance and gait deficits, investigation of the medications used, guidance on the choice of appropriate footwear and investigation of environmental factors form viable alternative approaches that can optimize the reduction of modifiable risk factors for recurrent falls.^31,32^ A care plan developed between the professional and the elderly individual, regarding prevention of falls, can also be an important facilitator for gaining knowledge of their clinical history, as well as for the activities and interventions that can be implemented, according to their interests and needs, while always aiming to heed the uniqueness of each case.^33^

In terms of clinical variables, the only significant difference among the groups was in relation to polypharmacy, although other clinical conditions can also increase the risk of falls.^30^ The consensus in the literature is that polypharmacy increases the risk of falls even after excluding other associated factors. Moreover, social factors and demographic differences need to be taken into consideration, along with the type and dose of medications, when interpreting this association.^1,33^ Visual impairment is another variable that can increase the possibility of falls among older people.^34^ Thus, altered constructional praxis may reflect not only impaired executive functioning, but also an individual’s capacity for visual perception. Therefore, one limitation of the present study was that this variable may not have been evaluated adequately, considering that the results may have been influenced by some unidentified visual deficit.

Other limitations of this study should be considered, such as the way in which falls were assessed and classified, which was based on a simple question that was subject to recall bias. Moreover, the cross-sectional design precluded establishment of a causal relationship between the variables studied.

The strength of this study was its use of more specific memory tests, which may have favored the finding regarding the association between memory and falls. Nonetheless, longitudinal studies are needed in order to confirm this finding and evaluate this domain specifically. Moreover, older people who experienced a single fall in the previous 12 months exhibited no evidence of poorer cognitive performance, in comparison with non-fallers, given that these two groups did not show any significant differences in terms of cognition.

## CONCLUSION

The prevalences of falls and cognitive impairment among the elderly are factors of concern for researchers and healthcare professionals. We found that elderly people who suffered recurrent falls had impaired memory and executive functioning, compared with non-fallers and sporadic fallers. Furthermore, having suffered only one fall in the last year did not seem to be associated with worse performance in cognitive tests. Hence, interventions need to be planned so that elderly sporadic fallers will not suffer more falls and thus evolve to become recurrent fallers.
